# Placement of ureteric stents to address postoperative urinary leakage in calyceal diverticulum with partially duplicated ureters resembling a renal cyst: A case report

**DOI:** 10.1097/MD.0000000000043861

**Published:** 2025-08-15

**Authors:** Hong Liu, Long Huang, Fan Liu, Dong liang Liu, Yan Xu, Lin Wang, Wen Ou

**Affiliations:** a Department of Urology, 363 Hospital, Chengdu, Sichuan Province, China.

**Keywords:** calyceal diverticulum, case report, ureteral duplication, ureteroscopic ureteral stent placement, urine leakage

## Abstract

**Rationale:**

Renal pelvic diverticulum accounts for fewer than 5% of renal cystic lesions. Its coexistence with a duplicated renal system is exceedingly rare, posing significant diagnostic and therapeutic challenges. These cases require differentiation from typical renal cysts and often necessitate a unique management approach.

**Patient concerns:**

A 57-year-old woman was initially misdiagnosed with a simple renal cyst and underwent laparoscopic cyst unroofing and decompression. Postoperatively, she developed persistent urinary leakage. Further investigation revealed a renal pelvic diverticulum associated with ureteral duplication.

**Diagnoses:**

Renal pelvic diverticulum combined with duplicated renal pelvis and ureter.

**Interventions:**

The patient underwent bilateral retrograde ureteral stent placement via flexible ureteroscopy, along with the insertion of a transurethral indwelling catheter to facilitate urinary drainage.

**Outcomes:**

At the 2-month follow-up, the patient was asymptomatic. Renal ultrasonography showed a 1 cm cystic lesion in the mid-pole of the right kidney without any perirenal fluid collection. Bilateral ureteral stents were successfully removed.

**Lessons:**

This case highlights the importance of distinguishing renal pelvic diverticula from simple cysts using advanced imaging modalities and demonstrates an effective strategy for managing postoperative complications such as urinary leakage.

## 1. Introduction

This study reports a rare case of a calyceal diverticulum associated with with partially duplicated ureters, which was initially misdiagnosed as a renal cyst and subsequently resulted in postoperative urinary leakage.

## 2. Case report

**Figure 1.**
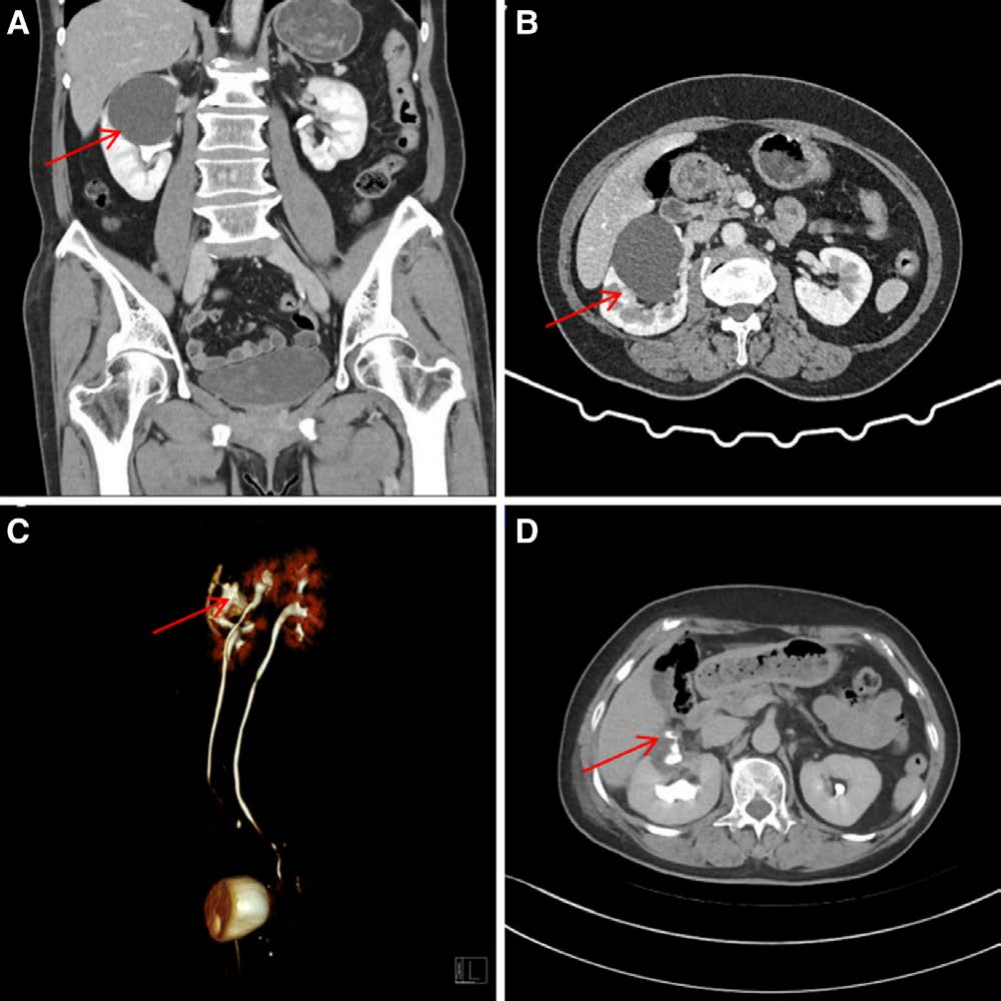
(A and B) Preoperative contrast-enhanced CT images demonstrate a hypodense cystic lesion in the mid-portion of the right kidney without contrast enhancement. (C and D) Postoperative CTU imaging findings. CT = computed tomography, CTU = CT urography.

A 57-year-old female patient was found to have a cystic mass in the right kidney, initially detected by ultrasound 2 years prior. The patient had no significant medical history, including hypertension, diabetes, or chronic nephritis. Routine laboratory tests, including a coagulation profile, liver and kidney function tests, electrolyte levels, blood glucose, lipid profile, and urinalysis, were all within normal limits. Contrast-enhanced abdominal CT revealed a 6 cm × 6 cm endophytic cyst located in the mid-portion of the right kidney, with no contrast enhancement observed during the excretory phase. The initial diagnosis was a right renal cyst, and laparoscopic cyst decortication was performed under general anesthesia (Fig. 1). During laparoscopic renal cyst decompression surgery, it was observed that the right renal cyst was located very close to the liver and protruded from the surface of the kidney, with a diameter of about 6cm. The cyst wall was thick, and the cyst fluid was clear when the cyst wall was cut open.

Postoperatively, the patient developed significant urinary leakage, with approximately 2000 mL of pale red fluid draining daily from the surgical site. Analysis of the drainage fluid indicated a creatinine level of 2217 µmol/L, confirming urine leakage. Further CT urography (CTU) revealed a cystic low-density area at the surgical site, with contrast enhancement during the excretory phase and extravasation outside the kidney. Additionally, a right duplex renal pelvis and ureter were identified. Consequently, the initial diagnosis of a renal cyst was revised to a dilated upper calyceal diverticulum.

**Figure 2.**
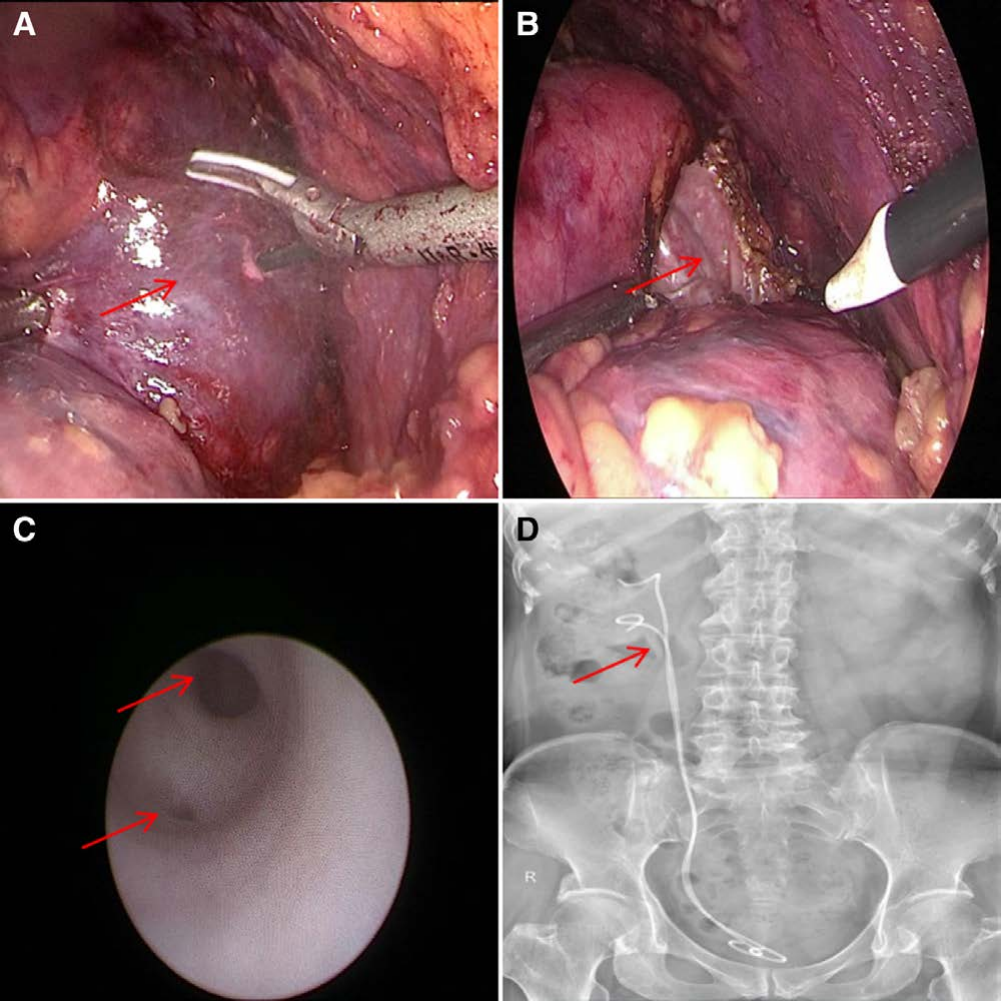
(A and B) Laparoscopic visualization of the cystic lesion in the right kidney. (C) Ureteroscopic view showing a bifurcation in the proximal segment of the right ureter. (D) Postoperative KUB radiograph following ureteral stent placement. KUB = kidney–ureter–bladder.

Flexible ureteroscopy, performed under intravenous anesthesia, revealed the right duplex renal pelvis and ureter, with no obvious rupture or opening in the diverticulum in either the upper or lower renal pelvis. Ureteral stents were placed in both ureters, and a Foley catheter was inserted to maintain low bladder pressure (Fig. 2).

**Figure 3.**
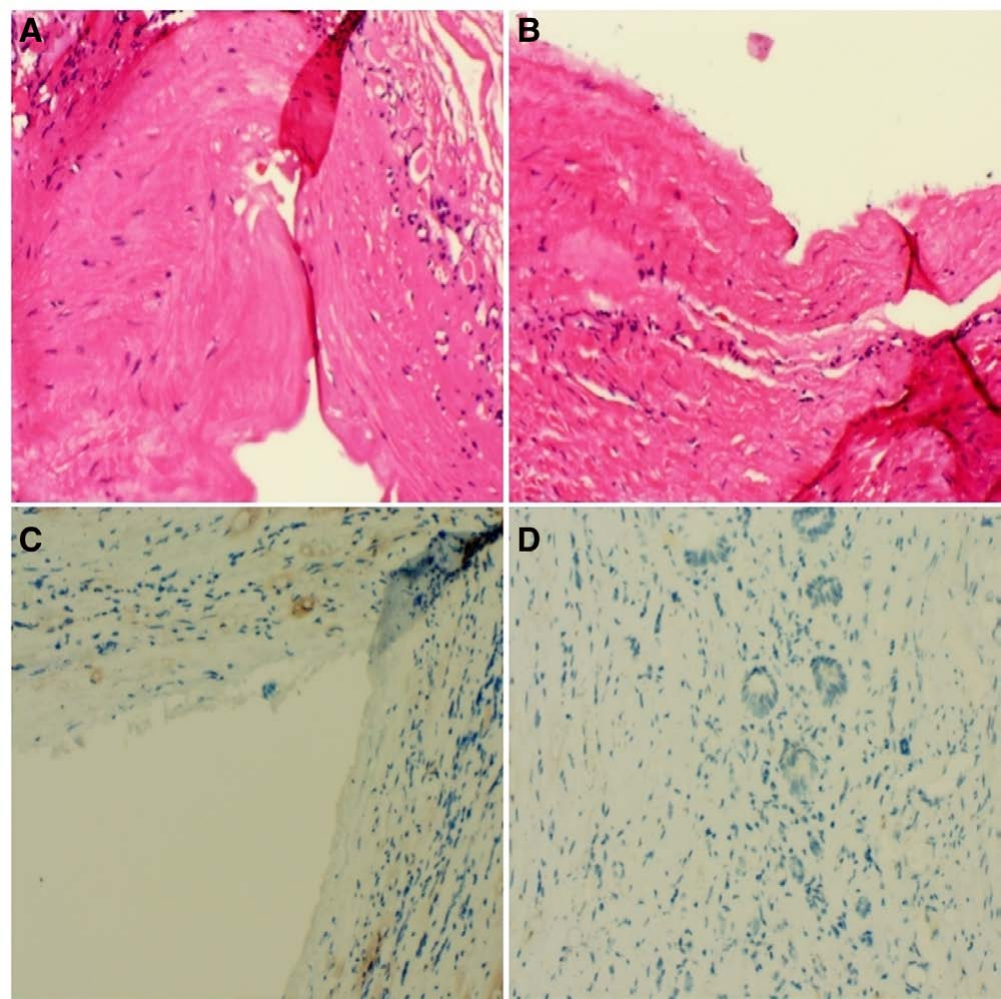
(A and B) Electron microscopy, a small number of epithelial cells with HE × 20 were observed. (C and D) immunohistochemistry showed negative CA9 HE × 40.

Urinary leakage ceased the day following stent placement. One week later, ultrasound examination showed a 3.4 cm × 2.0 cm anechoic area in the mid-portion of the right kidney, with clear boundaries and no evidence of hydronephrosis or perirenal fluid. The surgical drain was removed, and the Foley catheter was subsequently removed 2 weeks later during a follow-up visit. Pathological examination revealed a clearly defined cystic lesion measuring 8 cm × 6 cm in the gross specimen. Organ slices demonstrated glomerular and tubular tissues, and immunohistochemistry results were negative for CA9 (Fig. 3).

## 3. Results

Two months postoperatively, the patient remained asymptomatic, and a follow-up ultrasound revealed a 1 cm cystic area in the mid-portion of the right kidney without any perirenal fluid. Both ureteral stents were subsequently removed.

## 4. Discussion

Calyceal diverticula represent <5% of renal cystic lesions, and their occurrence alongside renal duplication is exceedingly rare, complicating both diagnosis and management.^[1,2]^ These diverticula are intrarenal cystic structures that communicate with the calyx or renal pelvis through a narrow isthmus, necessitating differentiation from endophytic renal cysts.^[3,4]^ Imaging modalities such as intravenous urogram (IVU), contrast-enhanced computed tomography (CT), and magnetic resonance imaging during the excretory phase can reveal contrast filling within the diverticulum.^[5]^ In the present case, the absence of contrast filling in the cystic mass on enhanced CT resulted in a misdiagnosis of a renal cyst. We propose that the narrow isthmus connecting the calyceal diverticulum to the renal pelvis may impede contrast filling during the typical 5 to 15 minute delay. Lin et al^[6]^ reported that extending the delay time from 10 to 60 minutes significantly enhances the sensitivity and accuracy of calyceal diverticula detection. Preoperative CT with a delay of more than 48 hours or retrograde pyelography with contrast injection followed by CT may represent more reliable strategies. Smyth and Romeiro et al^[7,8]^ suggested that in challenging preoperative diagnoses, a ureteral catheter can be placed, and methylene blue injected intraoperatively. If the cystic fluid turns blue, it confirms a calyceal diverticulum, indicating that cyst decortication should be avoided in favor of diverticulectomy with closure of the communication to the renal pelvis. For large renal cysts adjacent to the collecting system, the possibility of a calyceal diverticulum should be considered. Duplicated collecting systems may obscure communication between the diverticulum and the renal pelvis, complicating detection through routine preoperative imaging and intraoperative ureteroscopy. Zhang et al^[9]^ proposed that ultrasound-guided aspiration and creatinine measurement of the aspirated fluid can aid in differentiating suspected calyceal diverticula.

In this case, the patient experienced significant urinary leakage on the first postoperative day. Diagnosis of urine leakage was based on the elevated creatinine level in the drainage fluid, subsequently confirmed by CT urogram (CTU). Flexible ureteroscopy revealed no communication between the renal pelvis and the diverticulum, likely due to a small isthmus. Ureteral stents were placed to alleviate pressure in the renal pelvis, facilitating urine return to the collecting system and promoting adhesion and healing of the diverticular sinus. Given the duplex renal pelvis and ureter, stents were placed in both ureters to ensure adequate drainage. Ceylan et al^[10]^ reported a case in which oral desmopressin was employed to manage persistent urinary leakage following ureteral stent placement. Riggs et al^[11]^ described a case of a giant calyceal diverticulum in a child with high-output urinary leakage, successfully treated with ureteral stenting and fibrin glue injection. De Concilio and Aslan et al^[12,13]^ reported the use of gelatin sponge and N-butyl-2-cyanoacrylate for embolization in cases of persistent urinary leakage after partial nephrectomy, which may also be applicable to calyceal diverticula. In this instance, the duplex renal pelvis and ureter influenced the treatment strategy, with stents placed in both ureters for thorough drainage. Waingankar et al^[1]^ recommended that surgical planning for complex calyceal diverticula should consider anatomical variations, diverticulum location, and patient tolerance.

In conclusion, the diagnosis of calyceal diverticula should incorporate multiple imaging modalities, including prolonged CTU excretory phase imaging and retrograde pyelography, along with intraoperative exploration using methylene blue injection to enhance diagnostic accuracy. Treatment approaches should prioritize minimally invasive techniques while considering anatomical variations, diverticulum location, and patient tolerance.

## Author contributions

**Conceptualization:** Dongliang Liu.

**Investigation:** Fan Liu, Yan Xu, Lin Wang, Wen Ou.

**Writing – original draft:** Hong Liu.

**Writing – review & editing:** Long Huang.
